# Mitral valve prolapse: arrhythmic risk during pregnancy and postpartum

**DOI:** 10.1093/eurheartj/ehae224

**Published:** 2024-05-14

**Authors:** Avi Sabbag, Eivind W Aabel, Anna Isotta Castrini, Konstantinos C Siontis, Mikael Laredo, Jacky Nizard, Guillaume Duthoit, Samuel Asirvatham, Ojasay Sehrawat, Feddo P Kirkels, Philippe J van Rosendael, Roy Beinart, Moshe Rav Acha, Petr Peichl, Han S Lim, Christian Sohns, Raphael Martins, Jonaz Font, Nguyen N K Truong, Mette Estensen, Kristina H Haugaa

**Affiliations:** Sheba Medical Centre, Ramat-Gan, affiliated with the School of Medicine, Tel Aviv University, Tel Aviv, Israel; ProCardio Center for Research Based Innovation, Department of Cardiology, Oslo University Hospital, Rikshospitalet, and University of Oslo, Sognsvannsveien 20, 0372 Oslo, Norway; ProCardio Center for Research Based Innovation, Department of Cardiology, Oslo University Hospital, Rikshospitalet, and University of Oslo, Sognsvannsveien 20, 0372 Oslo, Norway; Department of Cardiovascular Medicine, Mayo Clinic, Rochester, MN, USA; Sorbonne Université, AP-HP, Groupe Hospitalier Pitié-Salpêtrière, Paris, France; Sorbonne Université, AP-HP, Groupe Hospitalier Pitié-Salpêtrière, Paris, France; Sorbonne Université, AP-HP, Groupe Hospitalier Pitié-Salpêtrière, Paris, France; Department of Cardiovascular Medicine, Mayo Clinic, Rochester, MN, USA; Department of Cardiovascular Medicine, Mayo Clinic, Rochester, MN, USA; Department of Cardiology, University Medical Centre Utrecht, Utrecht, The Netherlands; Department of Cardiology, University Medical Centre Utrecht, Utrecht, The Netherlands; Sheba Medical Centre, Ramat-Gan, affiliated with the School of Medicine, Tel Aviv University, Tel Aviv, Israel; Jesselson Integrated Heart Center, Shaare Zedek Medical Center, Jerusalem, Israel; Department of Cardiology, Institute for Clinical and Experimental Medicine, Prague, Czech Republic; Austin and Northern Health, University of Melbourne, Melbourne, Australia; Clinic for Electrophysiology, Herz- und Diabeteszentrum NRW, Ruhr-Universität Bochum, Bad Oeynhausen, Germany; LTSI, Rennes University Hospital, Rennes, France; LTSI, Rennes University Hospital, Rennes, France; Department of Interventional Cardiology, Medical University Center of Ho Chi Minh City, Ho Chi Minh, Vietnam; ProCardio Center for Research Based Innovation, Department of Cardiology, Oslo University Hospital, Rikshospitalet, and University of Oslo, Sognsvannsveien 20, 0372 Oslo, Norway; ProCardio Center for Research Based Innovation, Department of Cardiology, Oslo University Hospital, Rikshospitalet, and University of Oslo, Sognsvannsveien 20, 0372 Oslo, Norway; Department of Cardiology, Karolinska University Hospital, Stockholm, Sweden

**Keywords:** Arrhythmic mitral valve prolapse, Ventricular arrhythmia, Pregnancy, Mitral annular disjunction, Cardiomyopathy

## Abstract

**Background and Aims:**

Arrhythmic mitral valve prolapse (AMVP) is linked to life-threatening ventricular arrhythmias (VAs), and young women are considered at high risk. Cases of AMVP in women with malignant VA during pregnancy have emerged, but the arrhythmic risk during pregnancy is unknown. The authors aimed to describe features of women with high-risk AMVP who developed malignant VA during the perinatal period and to assess if pregnancy and the postpartum period were associated with a higher risk of malignant VA.

**Methods:**

This retrospective international multi-centre case series included high-risk women with AMVP who experienced malignant VA and at least one pregnancy. Malignant VA included ventricular fibrillation, sustained ventricular tachycardia, or appropriate shock from an implantable cardioverter defibrillator. The authors compared the incidence of malignant VA in non-pregnant periods and perinatal period; the latter defined as occurring during pregnancy and within 6 months after delivery.

**Results:**

The authors included 18 women with AMVP from 11 centres. During 7.5 (interquartile range 5.8–16.6) years of follow-up, 37 malignant VAs occurred, of which 18 were pregnancy related occurring in 13 (72%) unique patients. Pregnancy and 6 months after delivery showed increased incidence rate of malignant VA compared to the non-pregnancy period (univariate incidence rate ratio 2.66, 95% confidence interval 1.23–5.76).

**Conclusions:**

The perinatal period could impose increased risk of malignant VA in women with high-risk AMVP. The data may provide general guidance for pre-conception counselling and for nuanced shared decision-making between patients and clinicians.

## Structured Graphical Abstract

**Structured Graphical Abstract:**
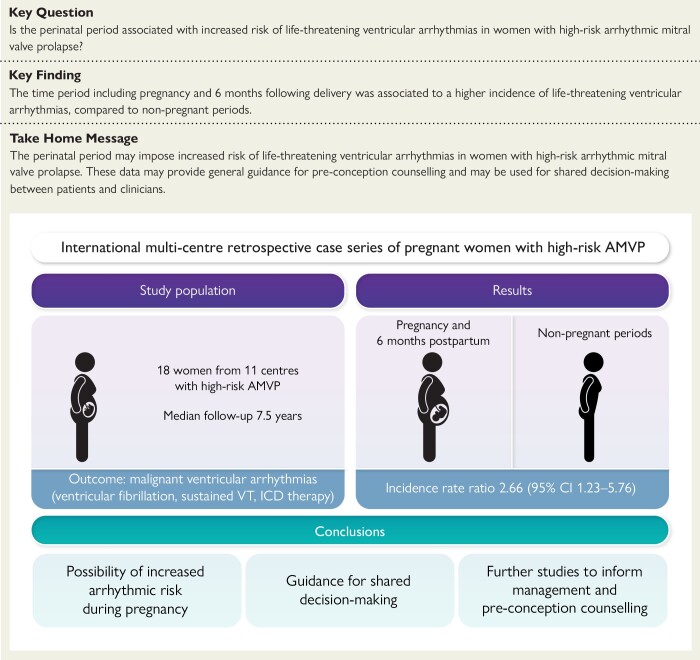
In this international multi-centre retrospective case series, we contacted 27 centres from four different continents for possible collaboration, and we included 18 women with AMVP from 11 centres. Malignant ventricular arrhythmia occurred 37 times, of which 18 (48%) occurred during pregnancy and 6 months postpartum. This period showed higher incidence rate of malignant VA, suggesting increased arrhythmic risk during the perinatal period. The findings from this study provide guidance for shared decision-making in high-risk AMVP women wanting to become pregnant. AMVP, arrhythmic mitral valve prolapse; CI, confidence interval; ICD, implantable cardioverter defibrillator; VA, ventricular arrhythmia; VT, ventricular tachycardia. The image graphics were generated using the Midjourney AI application, Midjourney.com.

**See the editorial comment for this article ‘Arrhythmic risk in young women with mitral valve prolapse: keep your eyes open but don't jump at every shadow', by K. Zeppenfeld and M. de Riva, https://doi.org/10.1093/eurheartj/ehae246.**

## Introduction

Mitral valve prolapse (MVP) is the most common valvular abnormality with a prevalence of 2%–3% in the general population.^1^ Until recently, the prognosis of MVP patients was believed to be largely defined by the severity of mitral regurgitation and subsequent left ventricular dysfunction. Nevertheless, the association between MVP and sudden cardiac death was established decades ago,^2,3^ and recent studies have identified a specific subpopulation of MVP patients signified by a high risk of sudden cardiac death independent of mitral regurgitation severity and left ventricular function.^4–6^ This newly defined clinical syndrome is termed arrhythmic MVP (AMVP).^7^

In the original reports of AMVP, young women were over-represented, and the occurrence of malignant ventricular arrhythmia (VA) during child-bearing age poses challenges. Pregnant women with structural heart disease have an increased risk of VAs, particularly during the last trimester.^8^ Data regarding women presenting with AMVP and malignant VA during pregnancy or the postpartum period are scarce.^8–10^ However, one study showed an increased risk of cardiac arrest during pregnancy in patients with MVP compared to women without MVP.^11^ The incidence of malignant VA during a normal pregnancy in unselected patients is very low,^12^ but these events may have dire consequences to both mother and foetus.^8,12,13^

In this study, we aimed to assess if pregnancy and the postpartum period are associated with a higher risk of malignant VA. Therefore, we formed an international multi-centre cohort of high-risk AMVP patients who had experienced pregnancy to compare incidence of VA in perinatal vs. non-pregnant periods.

## Methods

### Study design and patient population

The data that support the findings of this study are available from the corresponding author upon reasonable request. This study was designed as a retrospective international multi-centre case series with data collected according to a standard case report form. A study protocol was designed by the lead investigators and approved by the ethics committees of the Sheba Medical Center and Oslo University Hospital. A list of potential collaborators in Europe, Asia, America, and Australia was created, and each centre was approached from August 2022 to April 2023, in a pre-designed way by email that included an abbreviated study protocol inquiring about potential cases of women over the age of 18 years diagnosed with AMVP who experienced pregnancy, study aims, and inclusion/exclusion criteria. Centres that expressed an interest in participating received the full set of the pre-approved documents including the study protocol and electronic case report forms for data collection.

In accordance with the European Heart Rhythm Association consensus document, AMVP was defined as presence of MVP, combined with frequent and/or complex VA in the absence of any other well-defined arrhythmic substrates.^7^ To be eligible for study inclusion, patients also needed to have one of the following: (i) documented ventricular fibrillation (VF), sustained ventricular tachycardia (VT), or appropriate shock from an implantable cardioverter defibrillator (ICD) during pregnancy or within 6 months after delivery or (ii) history of VF, sustained VT, or appropriate ICD shock and with follow-up data during pregnancy. Patients were not eligible for inclusion when other well-defined arrhythmic substrates or structural heart diseases were present. Pregnancy and postpartum period were defined as 9 months before and 6 months after delivery. The start of follow-up was defined as the date of AMVP diagnosis or the date of first malignant VA if the malignant VA occurred before the AMVP diagnosis.

De-identified individual patient data were collected from each participating centre using standardized forms. We collected data on the women’s demographics, MVP diagnostic characteristics, pregnancy characteristics, Holter monitoring, electrocardiogram (ECG) QT interval measurements (mean, minimum, maximum, and corrected QT by Bazett’s formula), malignant VA manifestations, and long-term outcomes. The study complied with the Declaration of Helsinki. We only included women who provided authorization for use of their data for research, and each centre was responsible for their patients’ informed consent. Institutional review board approval was obtained at each participating institution.

### Ventricular arrhythmia

Premature ventricular complex (PVC) burden was defined as % of PVC beats per 24 h assessed by Holter monitoring. T-wave inversion was defined as present if seen in ≥two adjacent ECG leads. Malignant VA was defined as either aborted cardiac arrest, VF, appropriate shock from an ICD, or sustained VT (>100 b.p.m. lasting >30 s). We defined non-sustained VT as ≥3 consecutive ventricular beats at a rate >100 b.p.m. lasting <30 s. Multifocal PVCs were defined as the presence of ≥3 different PVC morphologies on Holter monitoring.

### Cardiac imaging

Mitral valve prolapse was diagnosed by echocardiographic or cardiac magnetic resonance imaging (CMR) before, during, or after pregnancy. Cardiac dimensions and functions were measured according to guidelines.^14–16^ We defined MVP as superior displacement ≥2 mm of any part of the mitral leaflet beyond the mitral annulus on echocardiography in the parasternal long-axis view.^14^ The mitral valve was defined as myxomatous if leaflet thickness was ≥5 mm. We defined mitral annular disjunction as ≥1 mm disjunction measured in end-systole from the left atrial wall–valve leaflet junction to the top of the left ventricular wall.^17^ Mitral regurgitation was evaluated by echocardiographic examination at MVP diagnosis. Late gadolinium enhancement (LGE) by CMR was also documented when present.^18^

### Statistical analysis

Continuous variables were presented as means with standard deviations or medians with interquartile ranges (IQRs) and categorical variables as absolute values with percentages. To explore whether the peripartum period was associated with increased incidence of malignant VA, we categorized each women’s follow-up in two periods, a perinatal period (encompassing pregnancy and 6 months postpartum) and a non-pregnancy period, and summed the number of malignant VA events within each period. Due to the possibility of repeated events within one patient, we performed a univariate mixed-effects Poisson regression with random effect on individual level, accounting for time in each period. For ECG QT interval measurements, we used univariate mixed-effects linear regression with random effect on individual level (Stata/SE v16.1, StataCorp LLC, College Station, TX, USA). Due to the low number of patients, no statistical hypothesis testing was performed and no *P*-values were reported.

## Results

### Patient population

**Figure 1:**
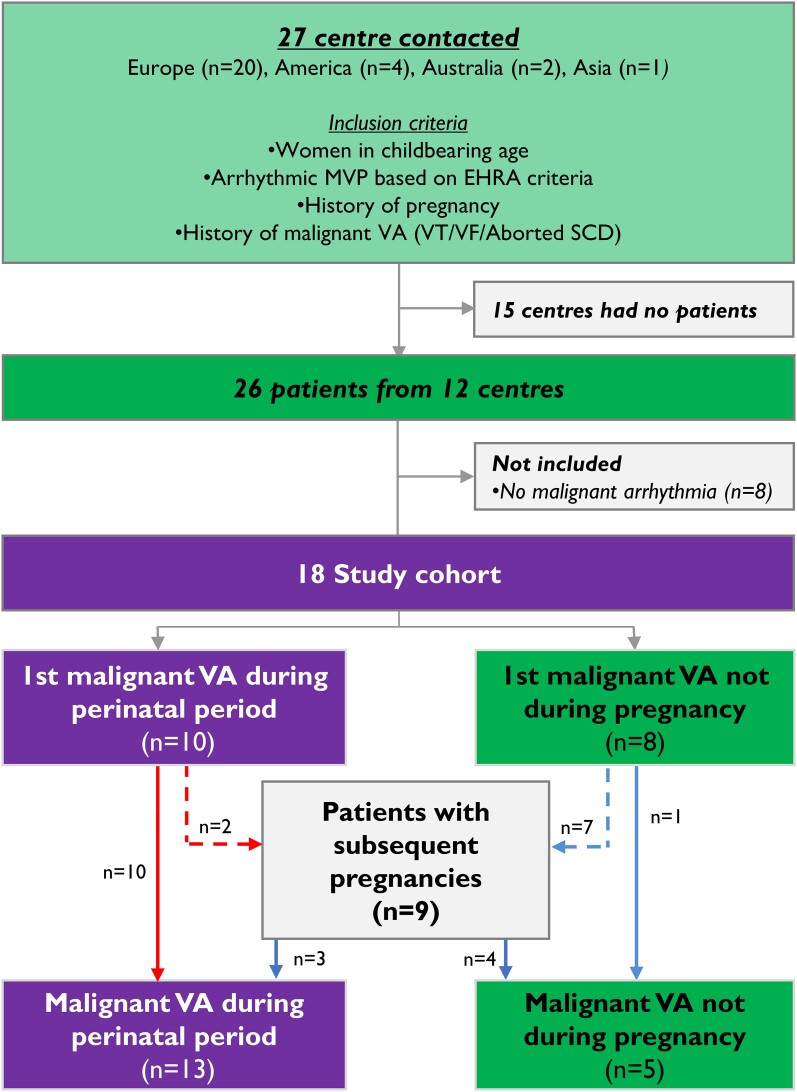
Study recruitment and inclusion chart. We contacted 27 centres from four different continents for possible collaboration on this case series, and we received 26 women with arrhythmic mitral valve prolapse from 12 centres. Eight women were not included due to not meeting predefined inclusion criteria. Thus, 18 women were included in this study

Out of the 27 centres approached for potentially eligible women, 12 centres had eligible women. We collected data on 26 women (range 1–5 per centre; *Figure 1*, Supplementary data online, *Table S1*). After detailed review, 8 women did not fulfil inclusion criteria and were excluded (all had only non-sustained VA). Thus, 18 eligible women [median age at AMVP diagnosis 24 (IQR 19–32) years] were included in this study, with median follow-up of 7.5 years (IQR 5.8–16.6) after AMVP diagnosis [*Table 1*; median 4.5 years (1.7–13.0) from AMVP diagnosis to first pregnancy and median 4.6 years (IQR 2.4–9.5) follow-up after first pregnancy]. Median age at first pregnancy after AMVP diagnosis was 29 years (IQR 27–34).

**Table 1:** Clinical characteristics of study participants

Characteristics	Total (*n* = 18)
**Clinical characteristics**	
Age at MVP diagnosis, years [IQR]	24 [19–32]
Number of pregnancies, *n* [IQR]	1.5 [1–2]
Pregnancy complicated by malignant VA, *n* (%)	13 (72)
Obstetrical complications, *n* (%)	3 (17)
Mode of delivery	
Vaginal delivery, *n* (%)	6 (55)
Caesarean section, *n* (%)	3 (27)
Other^a^, *n* (%)	2 (18)
T-wave inversions on ECG, *n* (%)	7 (41)
Aborted cardiac arrest, *n* (%)	15 (83)
Monomorphic VT, *n* (%)	5 (28)
Recurrent VT/VF, *n* (%)	10 (56)
During pregnancy, *n* (%)	4 (22)
Cardiac symptoms	
Cardiac syncope, *n* (%)	7 (39)
Unexplained syncope, *n* (%)	2 (11)
Anti-arrhythmic drugs during pregnancy	12 (67)
Beta-blocker, *n* (%)	7 (39)
Flecainide, *n* (%)	1 (6)
Flecainide and beta-blocker, *n* (%)	3 (17)
Quinidine and beta-blocker, *n* (%)	1 (6)
Ablation for ventricular arrhythmia, *n* (%)	6 (33)
PVC-triggering VF ablation, *n* (%)	6 (100)
VT ablation, *n* (%)	2 (33)
**Cardiac imaging**	
MVP, *n* (%)	18 (100)
Barlow, *n* (%)	13 (76)
Fibroelastic deficiency, *n* (%)	4 (24)
Inferolateral mitral annular disjunction (*n* = 13), *n* (%)	8 (62)
Left ventricular ejection fraction, %	57 ± 8
Mitral regurgitation	
No/trivial, *n* (%)	4 (22)
Mild, *n* (%)	6 (33)
Moderate, *n* (%)	7 (39)
Severe, *n* (%)	1 (6)
Late gadolinium enhancement (*n* = 10)	
No, *n* (%)	7 (70)
Left ventricular myocardium only, *n* (%)	2 (20)
Left ventricular papillary muscles only, *n* (%)	0 (0)
Both, *n* (%)	1 (10)
**First reported Holter monitoring (*n* = 15)**	
Performed during pregnancy, *n* (%)	3 (20)
PVC burden, % per 24 h [IQR]	5.0 [0.5–9]
Multifocal PVCs, *n* (%)	10 (67)
PVC couplets, *n* (%)	13 (87)
Non-sustained VT, *n* (%)	10 (67)
Sustained VT, *n* (%)	0 (0)

MVP, mitral valve prolapse; MVA, malignant ventricular arrhythmia; PVC, premature ventricular complex; VT, ventricular tachycardia; VF, ventricular fibrillation.

^a^One missed abortion and one terminated pregnancy.

All the women presented with aborted sudden cardiac death due to VF that required resuscitation and defibrillation. Most women had the myxomatous type of MVP, and moderate–severe mitral regurgitation was present in 8 patients (45%). T-wave inversions in the inferolateral leads were present in 7 women (41%). Data regarding mitral annular disjunction was only provided in 13 patients, of which 8 patients (62%) had inferolateral mitral annular disjunction. Cardiac magnetic resonance imaging was performed in 10 patients (56%), of which 1 was performed during pregnancy, 3 were performed in the postpartum period, and 6 were performed in the non-pregnancy period. Only 3 patients (30%) had LGE, of which 2 had left ventricular LGE only, while 1 had both left ventricular and papillary muscle LGE (*Table 1*).

Holter monitoring was reported in 15 women (83%), and the median PVC burden was 5.0% per 24 h (IQR 0.5–9.0); 10 women (67%) had multifocal PVCs and 10 (67%) had non-sustained VT. Premature ventricular complex–triggering VF ablation was performed in 6 women (33%), and 2 (11%) underwent simultaneous VT ablation.

### Arrhythmic mitral valve prolapse diagnosis and malignant ventricular arrhythmias

**Figure 2:**
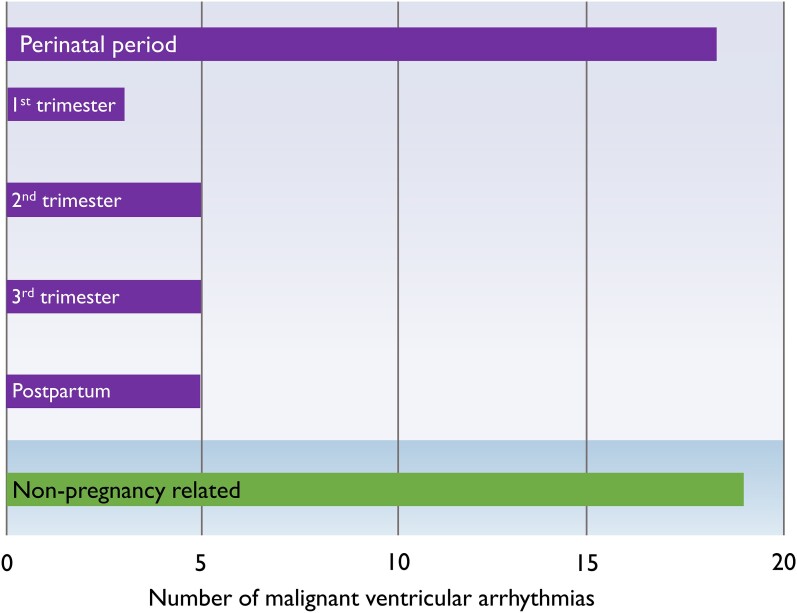
Distribution of 37 malignant ventricular arrhythmias during the perinatal and non-pregnancy periods. Thirty-seven malignant ventricular arrhythmias occurred in the 18 women in this study, of which 18 occurred in the perinatal period in 13 patients. The perinatal period had higher incidence rate of malignant ventricular arrhythmias

There were 37 malignant VAs during the study period. In 6 (27%) women, the first malignant VA occurred before their first-ever pregnancy, 8 (36%) women had their first malignant VA during pregnancy or in the postpartum period, while 5 (28%) patients had malignant VA in non-pregnant periods only (*Figure 1*). Eighteen (49%) of the 37 malignant VAs occurred during the perinatal period in 13 (59%) women (*Figure 2*). The event rate was 0.15 events per patient-year in the non-pregnancy period and 0.59 per patient-year in the perinatal period. When subdividing the perinatal period, the event rate was 0.66 per patient-year during pregnancy and 0.48 per patient-year in the postpartum period.

The perinatal period had an increased incidence rate of malignant VA compared to non-pregnancy periods in the same women [univariate incidence rate ratio (IRR) 2.66, 95% confidence interval (CI) 1.23–5.76]. However, upon examining the perinatal period through sub-analyses, we found that the postpartum period (IRR 2.54, 95% CI 0.93–6.94) and pregnancy (IRR 3.56, 95% CI 1.59–7.98) had both increased incidence rate of malignant VA with a numerically slightly higher risk during pregnancy. We found no difference in VA incidence between the different trimesters (*Figure 2*). Most malignant VAs were due to VF (*n* = 32, 87%), whereas only 5 (13%) events were sustained monomorphic VT, occurring in 5 unique women. None of the women died from the malignant VA.

### Pregnancy characteristics

**Figure 3:**
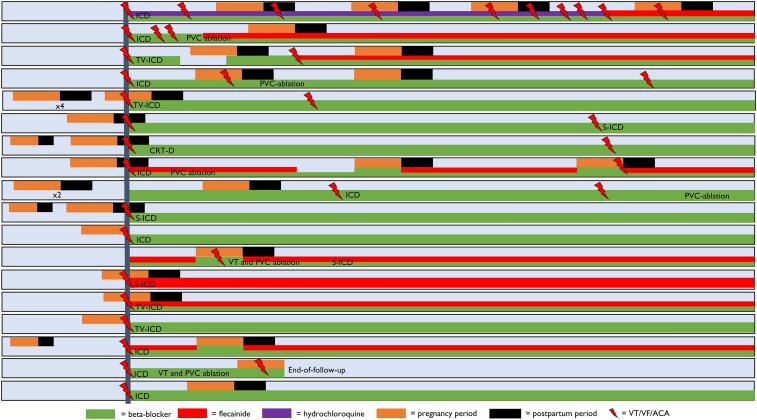
Individual level data on 18 women with arrhythmic mitral valve prolapse and pregnancy. A schematic overview of the 18 women included in this study (represented with one line) and the relationship between malignant ventricular arrhythmia (lightning symbol) occurring during pregnancy (orange bar), 6 months following delivery (black bar), or non-pregnant periods (light blue bar). Anti-arrhythmic therapies included beta-blockers (green bar), flecainide (red bar), and hydroxychloroquine (purple bar). The vertical line represents the time of arrhythmic mitral valve prolapse diagnosis. Malignant ventricular arrhythmia was reported 37 times, of which 13 women had malignant ventricular arrhythmia during the perinatal period

By definition, all included women experienced at least one pregnancy, and nine women experienced repeated pregnancies. Eight women (44%) were diagnosed with AMVP during the perinatal period due to malignant VA (*Figure 3*). One woman experienced a resuscitated cardiac arrest and miscarriage in gestational week 14, and another pregnancy was terminated at gestational week 8 due to multiple events of cardiac arrests requiring resuscitation. Despite malignant VA occurring during pregnancy, few obstetrical complications occurred, and the majority of deliveries were vaginal. Twelve (67%) women received prophylactic drug therapy during pregnancy, with beta-blockers most commonly used (*Table 1*). Only 9 of the 18 women had echocardiographic data during pregnancy. Of these, only 2 showed a change in mitral regurgitation severity and were both increased from mild to moderate. Only 1 woman had moderate–severe mitral regurgitation during pregnancy.

We collected 67 ECGs (42 during non-pregnancy and 25 during pregnancy) from our 18 women. The mean QTc during pregnancy was 425 ± 43 ms and during non-pregnancy 432 ± 24 ms. The QTc interval was slightly longer during pregnancy, but with wide CIs [6 ms (95% CI −15 to 28)]. QT dispersion was numerically lower during pregnancy [−11 ms (95% CI −22 to 0)]. QTc was similar in women that experienced malignant VA during pregnancy compared to women that experienced malignant VA during non-pregnancy [0 ms (95% CI −35 to 36)]. QT dispersion was lower in women that experienced malignant VA during pregnancy [−23 ms (95% CI −45 to 0)].

### Management strategies of malignant ventricular arrhythmias during pregnancy

An overview of the anti-arrhythmic therapies used can be found in *Figure 3* and Supplementary data online, *Figure S1*. Most patients continued the anti-arrhythmic medication used prior to pregnancy, and the dosage was not changed. Prior flecainide treatment was discontinued during pregnancy in three women, while three women continued prior use of flecainide during pregnancy with similar dosage. Prior beta-blocker use was continued during pregnancy in almost all women, except one that discontinued beta-blocker prior to pregnancy due to experiencing several miscarriages possibly related to beta-blocker use. Among the 10 women with a first malignant VA during the perinatal period, treatment of beta-blocker alone was started in 6, flecainide was started in 3 (of which 1 without beta-blocker), and 1 patient underwent VT and PVC ablation during pregnancy. Additionally, ICD implantation was performed in 9 women during the perinatal period, while implantation was postponed until after pregnancy in the woman undergoing ablation.

In patients with ICD who experienced ICD shock, no change in medication or dosage was performed.

## Discussion

This is the first report evaluating the burden of VA events associated with the perinatal period in women with AMVP. In this multi-centre international case series of women with AMVP and a high-risk arrhythmic phenotype, we observed an increased incidence rate of malignant VAs during the perinatal period as compared to non-pregnancy periods in the same women (*Structured Graphical Abstract* ). This finding may be important in pre-conception counselling of women with AMVP, in decisions on anti-arrhythmic medication during pregnancy, and ultimately in decisions on primary prevention ICD implantations. Importantly, most pregnancies progressed to term with a low number of obstetrical complications.

By design, our cohort was very specific, focusing on young women of child-bearing age meeting the European Heart Rhythm Association criteria for AMVP.^7^ Our inclusion criteria yielded a high-risk group, where all patients had a history of malignant VA and even aborted sudden cardiac death. Risk factors in this cohort were similar to other high-risk AMVP groups,^4,6^ such as history of syncope, T-wave inversions, myxomatous MVP, and mitral annular disjunction. In contrast, our cohort had a surprisingly low proportion of women with LGE on CMR, though CMR was performed in only a subset of participants. Previous studies have reported LGE in 28%–36%^19,20^ with higher rates in patients with more severe mitral regurgitation.^20^ Our lower proportion of LGE may be explained by the young age of our participants, representing early stages of local remodelling, not yet progressing into replacement fibrosis. The fact that life-threatening arrhythmias occurred in several women without LGE is an important reminder that the absence of LGE is not necessarily a low-risk feature. Interestingly, five women in our study experienced monomorphic VT, where LGE is thought to represent the main substrate.

### Pregnancy and the risk of arrhythmia

This study showed a seemingly increased incidence rate of malignant VA during the perinatal period compared to non-pregnancy in women with AMVP, with 2.5-fold higher incidence rate. This is in line with a prior observational study showing increased risk of maternal and foetal adverse outcomes during 23 000 pregnancies in women with MVP, with four-fold increased odds of cardiac arrest.^11^ Pregnancy, rather than the postpartum period, seemed to constitute the period of highest risk in our study. Whether there is a true difference in risk between these periods remain to be explored in larger studies. However, even though the risk increases during pregnancy and the postpartum period, the absolute incidence rate of malignant VAs during the perinatal period in AMVP women remains low, as inferred by the very low number of cases found in 27 large medical centres.

The risk of malignant VA increases during the perinatal period in women with a variety of cardiac diseases, including structural heart disease and primary electrical disease.^8,21^ The mechanisms are multifactorial and include altered hormonal levels, haemodynamic changes, and an altered autonomic balance with predominantly sympathetic drive at rest.^22^ None of these alterations have been studied specifically in MVP, yet it may be reasonable to assume that some of these pathophysiological processes play a role in the AMVP population as well.

It has been postulated that mechanical traction and myocardial stretch caused by the prolapsing leaflet may be central to the arrhythmogenesis of MVP.^23^ Myocardial stretch may lead to a decrease in resting diastolic membrane potential, shortening of the action potential duration, and the development of stretch-induced early afterdepolarizations.^24^ These effects tend to be more pronounced in areas that are subjected to more traction,^25^ leading to action potential heterogeneities setting the stage for functional re-entry circuits. Pregnancy is associated with an increased effective circulating blood volume of up to 50%, possibly further accentuating these changes leading to a further increase in the risk of arrhythmia. Importantly, this mechanism may be independent of fibrotic substrates.^26^

Female sex hormones, especially oestrogen, affect cardiac repolarization causing QT prolongation, which is an expected observation in normal pregnancy.^21,27,28^ Interestingly, patients with AMVP tend to have longer QT than unselected patients with MVP,^29^ suggesting a similar vulnerability to the changes in the hormonal levels, possibly influencing the risk of VA.^21^

Pregnancy is also associated with altered pharmacokinetics of many medications^30^ in a progressive manner throughout pregnancy stages, which could explain the increased incidence of arrhythmic events seen in our study. Of interest, pregnancy is associated with increased activity of CYP2D6, which metabolizes many anti-arrhythmic drugs, including metoprolol, flecainide, and mexiletine. However, despite lower plasma concentrations of metoprolol during pregnancy, the chronotropic effect seems to be greater during pregnancy,^31^ suggesting altered sensitivity to metoprolol due to predominant sympathetic drive at rest during pregnancy.

### Management implications

Thus far, guidance for the management of pregnant women with AMVP has been based on a limited number of case reports and expert opinions. Our findings have important implications that can inform the management of this complex clinical scenario and generate initial evidence to guide pre-conception counselling and arrhythmia surveillance during pregnancy. Women with AMVP who wish to become pregnant and their physicians should be aware of a potentially increased arrhythmic risk as observed in our study. In response, intensified arrhythmia surveillance approaches may be considered, aiming to capture precursors of malignant VAs such as high-risk PVCs and non-sustained VT. Finally, further studies should explore if a lower threshold for primary preventive ICD implantation should be considered in high-risk patients as part of pre-conception counselling. Furthermore, it is unknown whether surgical repair or replacement of severe mitral regurgitation decreases arrhythmic risk in AMVP patients. Whether prophylactic anti-arrhythmic drug therapy during pregnancy may be advisable in AMVP patients is yet to be explored. Both treating cardiologists and pregnant women may be reluctant towards anti-arrhythmic drugs due to possible risk to the foetus and mother. However, metoprolol, flecainide, and quinidine have long records of safety during pregnancy with minimal teratogenic effects and low foetal and maternal risk.^32^ These complex decisions are best made under the care of clinicians with expertise in AMVP in collaboration with maternal–foetal medicine in tertiary care centres. While the retrospective case series nature of our report does not allow management recommendations, these data may provide general guidance for nuanced shared decision-making between patients and clinicians.

### Study limitations

This is a retrospective observational study with inherent limitations with a low number of women affecting the robustness of the results. In order to avoid selection bias, we used a strict set of inclusion criteria resulting in a relatively homogenous high-risk cohort. We minimized differences in the intensity of medical care by beginning the follow-up at the time of AMVP diagnosis.

We included only women with AMVP patients that presented with malignant VA and were pregnant at least once. These narrow inclusion criteria reduce the applicability of these results to other AMVP populations. Similarly, our study design does not allow evaluation of the true attributable risk of the perinatal period. Furthermore, due to the retrospective nature, all women had to survive their index event to allow for diagnosis, extensive evaluation, and follow-up in order to be eligible for inclusion in this cohort. Nevertheless, our study design may be subject to recall bias, as healthcare providers may be more prone to remember pregnant patients with life-threatening events compared to non-pregnant patients. However, the design using each woman as her own control by comparing periods of pregnancy to non-pregnant periods may partly overcome this limitation.

Some of the women in this study did not undergo CMR or Holter monitoring precluding any concrete conclusions regarding these aspects of the study. We were further limited in studying pregnancy related changes in function of the mitral apparatus and their association with arrhythmic risk, as repeat echocardiographic evaluation was available for only a few women in this study.

However, we believe that the design of our study using the women as their own controls limits the impact of recall bias and selection bias. Moreover, comparing events during pregnant and non-pregnant periods may partly overcome some of the inherent limitation. Finally, we do not have comparative echocardiographic, ECG, and Holter monitoring data during pregnancy and outside of pregnancy periods for the included patients.

## Conclusions

The perinatal period could impose increased risk of malignant VAs in women with high-risk AMVP. Our data, although based on a small number of highly selected women that experience malignant VAs, may provide general guidance for pre-conception counselling and for nuanced shared decision-making between patients and clinicians. Further studies are needed to inform management of this population during pregnancy and in pre-conception counselling.

## Supplementary data

Supplementary data are available at *European Heart Journal* online.

## Supplementary Material

ehae224_Supplementary_Data

## Declarations

### Data Availability

The data that support the findings of this study are available from the corresponding author upon reasonable request.

### Pre-registered Clinical Trial Number

None supplied.
