# Supplementary material for: Mitral valve prolapse: arrhythmic risk during pregnancy and postpartum
Source: Eur Heart J. 2024 May 14;45(20):1831–9. doi: 10.1093/eurheartj/ehae224 (PMC11129793; doi:10.1093/eurheartj/ehae224)
Supplement: ehae224_Supplementary_Data [file ehae224_supplementary_data.docx]

**Supplemental Table 1. Number of reported patients per center contacted as part of the data collection.**

| **Country** | **Center** | **Number of patients reported** | **Number of patients included** |
| --- | --- | --- | --- |
| Israel | Sheba | 3 | 2 |
|  | Tel Aviv | 0 | 0 |
|  | Jerusalem | 1 | 1 |
|  | Haifa | 0 | 0 |
|  | Belinson | 0 | 0 |
| France | Rennes University Hospital | 2 | 2 |
|  | Paris | 8 | 3 |
|  | Toulouse | 0 | 0 |
|  | Bordeaux | 0 | 0 |
|  | Cannes | 0 | 0 |
|  | Caen | 0 | 0 |
| The Netherlands | Maastricht | 0 | 0 |
|  | Utrecht | 3 | 2 |
| Germany | Leipzig | 0 | 0 |
|  | Bad Oeynhausen | 1 | 1 |
| Czech Republic | Prague | 1 | 1 |
| Norway | Oslo University Hospital | 2 | 2 |
| Sweden | Karolinska University Hospital | 1 | 1 |
| Italy | Padua | 0 | 0 |
| Belgium | Brussel | 0 | 0 |
| United States of America | Cleveland Clinic | 0 | 0 |
|  | Penn | 0 | 0 |
|  | Mayo Clinic | 2 | 2 |
|  | Rochester, NY | 0 | 0 |
| Australia | Adelaide | 0 | 0 |
|  | University of Melbourne | 1 | 0 |
| Vietnam | Medical University Center of Ho Chi Minh City | 1 | 1 |

**Supplemental Figure 1.** **Individual level data centered on first pregnancy.**


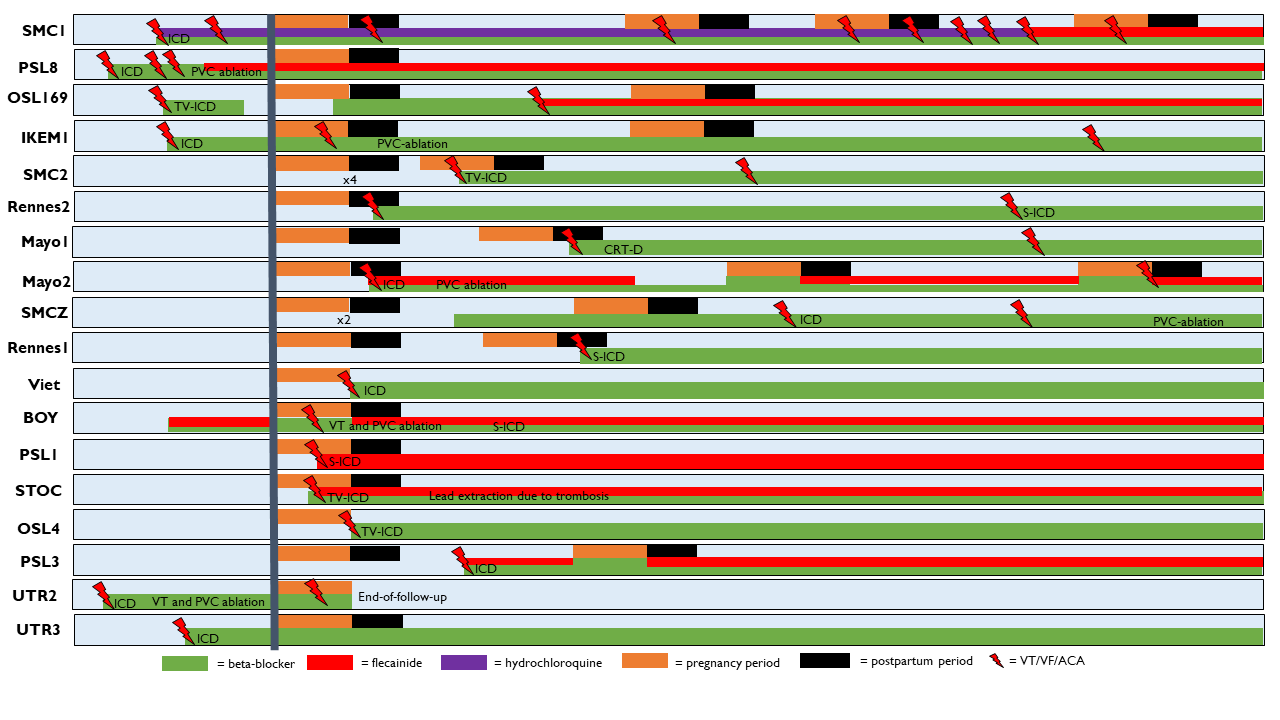


A schematic overview of the 18 women included in this study (represented with one line) and the relationship between malignant ventricular arrhythmia (red lightning) occurring during pregnancy (orange bar), 6 months following delivery (black bar), or non-pregnant periods (light blue bar). Antiarrhythmic therapies included beta-blockers (green bar), flecainide (red bar) and hydrochloroquine (purple bar). The vertical line represents the time of first pregnancy. Malignant ventricular arrhythmia was reported 37 times, of which 13 women had malignant ventricular arrhythmia during the perinatal period.
